# Draft genome of *Thermoanaerobacter thermohydrosulfuricus* strain AK152, a novel thermophilic and anaerobic bacterium isolated from a hot spring in Iceland

**DOI:** 10.1128/mra.01175-23

**Published:** 2024-08-28

**Authors:** Clay A. Abraham, Kevin M. Bradley, Sean Michael Scully, Johann Orlygsson, Derek Dube, Steven A. Benner

**Affiliations:** 1 Foundation for Applied Molecular Evolution, Alachua, Florida, USA; 2 Department of Biology, University of Saint Joseph, West Hartford, Connecticut, USA; 3 Department of Natural Resource Science, University of Akureyri, Akureyri, Iceland; Indiana University—Bloomington, Bloomington, Indiana, USA

**Keywords:** *Thermoanaerobacter*, genomes, *Thermophilic anaerobe*

## Abstract

We present the draft genome of the bacterium *Thermoanaerobacter thermohydrosulfuricus* strain AK152, a thermophilic, endospore–spore-forming, anaerobe isolated from a hot spring in Grensdalur, in Southwestern Iceland. This assembled genome will lay the foundation for identifying the carboxylic and amino acid fermentation pathways, suggesting biotechnological applications for this strain.

## ANNOUNCEMENT

The *Thermoanaerobacter* genus is comprised of strict anaerobic thermophilic heterotrophic bacteria that have been detected in multiple extremophilic environments (1–5). *Thermoanaerobacter thermohydrosulfuricus* strain AK152 (AK152) was isolated by serial dilutions of sediment from a hot spring in Grensdalur, Iceland. AK152 is ethanologenic and is exceptional in its ability to reduce multiple carboxylic acids to their corresponding alcohols and degrade a broad range of carbohydrates, yielding ethanol as the dominant end product (6).

AK152 was cultivated in basal mineral media with a 1.0 liquid-to-gas phase ratio and N_2_ gas (<5% ppm O_2_) for 48 h at 65°C (7, 8). Isolated cell pellets were stored at −80°C. Sodium dodecyl sulfate (SDS) and phenol:chloroform:iso-amyl alcohol were used to lyse the cell pellet (9). To remove residual SDS, two rounds of ethanol precipitation were performed. Extracted DNA was resuspended in sterile dH_2_O and quantitated by a nanodrop spectrophotometer.

Library preparation and genomic sequencing of AK152’s DNA was performed by Beijing Genomic Institute (BGI) using DNBseq. A total of 0.24 µg of DNA was sent to BGI for library preparation with BGI’s Optimal DNA Library Prep Kit, which used PCR amplification. The MGISEQ-2000 platform with paired ends and 150 base pair (bp) reads sequenced the sample. BGI used the program SOAPnuke to obtain clean reads by removing adapter sequences, reads with 1% N content, reads below a quality of 20, and a minimum length of 150 bp (10). The cleaned raw data are listed in Table 1. The raw data from BGI were analyzed with FastQC (11). FastQC analysis found essentially no N reads, an average read quality of 39, and little sequence duplication.

**TABLE 1:** Analysis of *Thermoanaerobacter thermohydrosulfuricus* strain AK152 NGS and genome assembly data

Sequencing	Raw data	Assembly	SPAdes	SKESA
Clean Reads	4,055,209	Contigs	62	58
Clean Bases	1,216,562,700	Largest contig	229,593	181,799
Q20 (%)	96.62	N50	113,744	102,254
Q30 (%)	90.75	L50	8	9
GC (%)	35.09	GC (%)	33.83	33.78
		GeneMarkS (ORFs)	2,631	2,732
		PGAP (number of annotations)	811	2,154

The genome was assembled with two pipelines. The first was conducted with SPAdes version 3.15.4 using the isolate model (12). The second assembly method used the Read Assembly and Annotation Pipeline Tool (RAPT) version 0.5.4 suite of bioinformatics programs. RAPT includes the SKESA assembler version 2.5.0, ANI taxonomy identifier, NCBI Prokaryotic Genome Annotation Pipeline (PGAP), and CheckM (13–16).

The SPAdes and SKESA assemblies were both cross-referenced with QUAST version 5.2.0 for analysis (17). Additionally, each assembly was annotated with PGAP and the open reading frame finder GeneMarkS version 4.28 (18). Open reading frame locations (ORFs) were determined with GeneMarkS. Further, annotation of the assemblies was performed with PGAP. The metrics from the programs used to assemble and assess the genome are listed in Table 1.

Displayed in Table 1, the SPAdes assembly generated the largest contig and more contigs overall when compared to SKESA. However, after annotation with GeneMakS and PGAP, the SPAdes assembly produced fewer overall protein sequences and ORFs, with substantially fewer annotations made by PGAP. The PGAP annotation of the SKESA assembly identified over 2.5 × more protein sequences than the SPAdes assembly as observed in Table 1. From this, the SKESA assembly, while finding fewer contigs overall, produced a more comprehensive annotation of the AK152 genome.

## Data Availability

The assemblies in this article are deposited at DDBJ/ENA/GenBank under Biosample SAMN39271361 and under accession nos. JAZEUZ000000000 and JBAMAR000000000 for the SKESA and SPAdes assemblies, respectively. The raw data reads are deposited at SRA under Bioproject accession no. PRJNA1061494.
